# Not all (cells) who wander are lost: Upstream migration as a pervasive mode of amoeboid cell motility

**DOI:** 10.3389/fcell.2023.1291201

**Published:** 2023-11-02

**Authors:** Alexander Buffone, Daniel A. Hammer, Sarah Hyun Ji Kim, Nicholas R. Anderson, Ai Mochida, Dong-Hun Lee, Subham Guin

**Affiliations:** ^1^ Department of Biomedical Engineering, New Jersey Institute of Technology, Newark, NJ, United States; ^2^ Chemical and Materials Engineering, New Jersey Institute of Technology, Newark, NJ, United States; ^3^ Department of Bioengineering, University of Pennsylvania, Philadelphia, PA, United States; ^4^ Department of Chemical and Biomolecular Engineering, University of Pennsylvania, Philadelphia, PA, United States; ^5^ Department of Medicine, University of California San Diego, La Jolla, CA, United States; ^6^ Carisma Therapeutics, Philadelphia, PA, United States

**Keywords:** T-cells, leukocytes, inflammation, LFA-1, ICAM-1, hematopoietic stem cells, migration

## Abstract

Leukocytes possess the ability to migrate upstream—against the direction of flow—on surfaces of specific chemistry. Upstream migration was first characterized *in vitro* for T-cells on surfaces comprised of intracellular adhesion molecule-1 (ICAM-1). Upstream migration occurs when the integrin receptor α_L_β_2_ (also known as lymphocyte function-associated antigen-1, or LFA-1) binds to ICAM-1. LFA-1/ICAM-1 interactions are ubiquitous and are widely found in leukocyte trafficking. Upstream migration would be employed after cells come to arrest on the apical surface of the endothelium and might confer an advantage for both trans-endothelial migration and tissue surveillance. It has now been shown that several other motile amoeboid cells which have the responsibility of trafficking from blood vessels into tissues, such as Marginal zone B cells, hematopoietic stem cells, and neutrophils (when macrophage-1 antigen, Mac-1, is blocked), can also migrate upstream on ICAM-1 surfaces. This review will summarize what is known about the basic mechanisms of upstream migration, which cells have displayed this phenomenon, and the possible role of upstream migration in physiology and tissue homeostasis.

## Introduction

Leukocytes must traffic in and out of tissues to carry out numerous immune and effector functions. Blood borne cells, carried in a hydrodynamic flow, interact with the vascular endothelium through a multi-step process known as the leukocyte adhesion cascade (Ley et al., 2007). Leukocytes first tether and roll on the apical surface of endothelium, facilitated by selectins. Then integrins on leukocytes are activated through conformational changes and strongly bind to cognate ligands. Selectins and integrins act in synergy to facilitate leukocyte capture (Bhatia et al., 2003; Eniola et al., 2003). Leukocytes will spread and migrate on the apical surface of the endothelium to find junctions for transendothelial migration into tissues. The expression of leukocyte receptors depends on the leukocyte. For example, T-cells bear the integrin α_L_β_2_, also known as lymphocyte function-associated antigen-1 (LFA-1), which binds intracellular adhesion molecule-1(ICAM-1), as well as α_4_β_1_, or very late antigen-4 (VLA-4), which binds vascular cell adhesion molecule-1 (VCAM-1) (López-Hoyos et al., 1999). Neutrophils bear two beta-2 integrins—LFA-1 and macrophage-1 antigen (Mac-1, α_M_β_2_), which also binds to ICAM-1 (Heit et al., 2005). These integrin-ligand interactions dictate the dynamics and location of firm adhesion as well as migration of leukocytes in the vascular endothelium.

**FIGURE 1:**
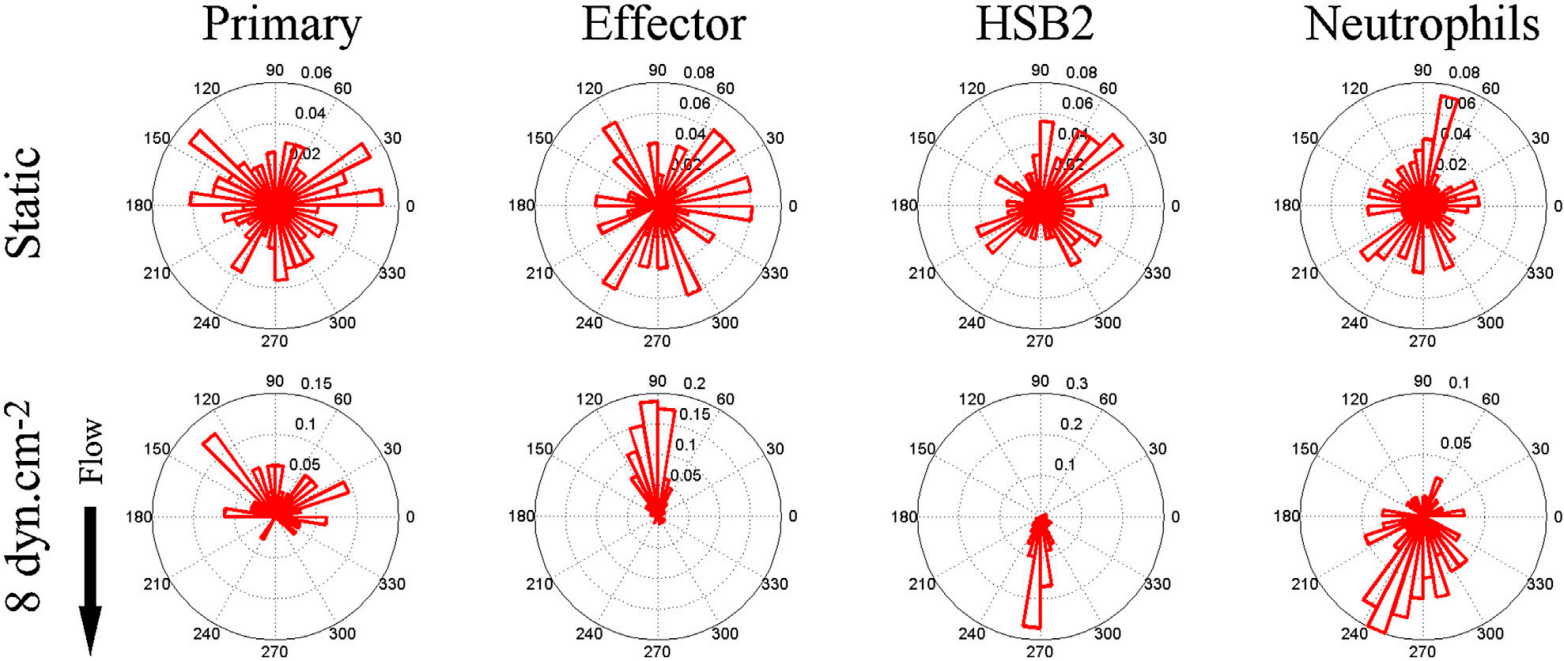
Angle histogram for one representative experiment in four different leukocyte cell populations (primary T cell, effector T cells, HSB2 T cells, and neutrophils) in static (*upper panels*) and flow (*lower panels*) conditions showing the distribution of *θ* in ≤40 angle bins. The length of each bin reflects the fraction of cells with a given angle that fall within a group. Under the flow condition, the shear stress was set to a value of 8 dyn cm^−2^ and the direction of the flow was along the *Y*-axis from top to bottom, as indicated by the arrow. The numbers of cells analyzed were respectively 192, 234, 148, and 134 in the static condition and 23, 73, 93, and 60 under the flow condition for primary T cells, effector T cells, HSB2 T cells, and neutrophils. Reproduced from Valignat et al., *Biophys J*, 2013 January 22; 104(2):322-31.

About a decade ago, Theodoly and colleagues pointed out that the migration of leukocytes on the apical endothelium occurs under a substantial shear flow (Valignat et al., 2013). In post capillary venules, adhesion and migration occur under shear stresses between 4 and 60 dynes/cm^2^ (Bhatia et al., 2003). Motivated by this observation, as well as the idea that leukocyte activity is modulated by shear stress and that lymphocytes had been observed migrating upstream in the vascular lumen during a murine model of autoimmune encephalitis (Bartholomaus et al., 2009), Theodoly et al. performed *in vitro* experiments on the migration of several types of leukocytes, including primary (naïve) T-cells, effector (activated) CD4^+^ T-cells, neutrophils, and a leukemic cell line (HSB2) on ICAM-1 surfaces under flow. Surfaces were prepared with a physisorbed ICAM-1-Fc in combination with the chemokine SDF-1a. Curiously, both naïve and effector T-cells migrated against the direction of flow on ICAM-1 surfaces, whereas neutrophils and HSB2 cells migrated downstream in the direction of flow (Figure 1).

There were several remarkable features of the upstream migration of T-cells. First, in the absence of flow, T-cells migrated randomly, but upon initiation of flow, T-cells rapidly oriented in the direction of flow (within 30 s) (Valignat et al., 2013). Second, effector T-cells oriented more avidly upstream than naïve T-cells (Valignat et al., 2013). Third, T-cells oriented upstream with greater fidelity when the shear stress increased (up to 60 dynes/cm^2^) (Valignat et al., 2013). And finally, HSB2 cells did not migrate upstream, despite having the same receptor profile as T-cells, and neutrophils also did not migrate upstream (Valignat et al., 2013). At the time, these results suggested that upstream migration is a phenomenon restricted to T-cells, especially effector T-cells. Ultimately, it would be shown that upstream migration is a much more pervasive mode of cell motility.

## Essential requirement for LFA-1 for upstream migration

Upstream migration is a fascinating phenomenon in its own right. Valignat and coworkers estimated the applied force on a migrating T-cell to be 0.6 nN at a shear stress of 60 dynes/cm^2^ (Valignat et al., 2013). Thus, T-cells must both withstand this force *and* generate a traction that can propel the T-cell against the direction of flow. The question is, what are the molecular origin and structural requirements for upstream migration?

Theodoly and coworkers advanced the idea that on ICAM-1 surfaces, T-cells take on a structure that supports upstream migration (Valignat et al., 2014). Roy *et al.* had observed that T cells on ICAM-1 surfaces have a broad, actin-rich lamellipod, whereas cells on VCAM-1 surfaces have a less pronounced lamellipod and reduced actin polymerization in the leading edge (Roy et al., 2020). 3D scanning microscopy showed that on ICAM-1 surfaces, T-cells have an adherent lamellipod but a detached uropod that vertically extends upward. It was suggested that this uropodial tail acts to passively steer the T-cell, like a “wind vane” (Valignat et al., 2014). This mechanism suggests the effect of receptor-ligand interactions is to maintain cell structure, without the need for internal signaling triggered by flow.

**FIGURE 2:**
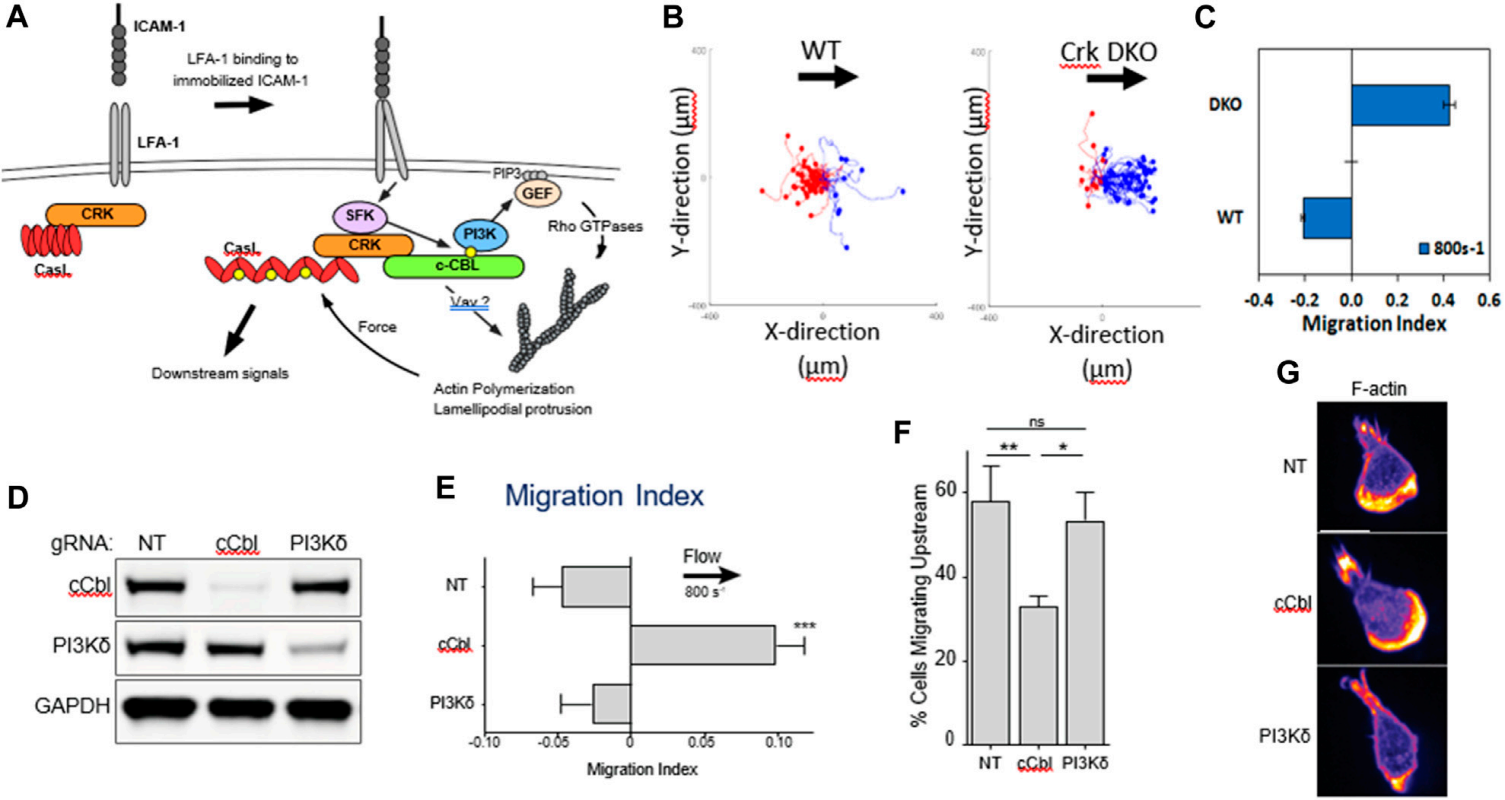
**(A)** Biochemical signals downstream of LFA-1 after engagement with ICAM-1. **(B)** Flow histograms and **(C)** Migration Indices of WT and Crk DKO mouse CD4^+^ T cells migrating on ICAM-1 surface at 800s^-1^ shear rate. **(D)** CD4^+^ T cells from Cas9-expressing mice were transduced with the indicated gRNAs, selected for 3 d in puromycin, and then lysed and immunoblotted for the indicated proteins. NT, non-targeting gRNA control. GAPDH was used as a loading control. **(E)** Migration index of T cells expressing the indicated gRNAs migrating on ICAM-1 under shear flow (shear rate 800 s^−1^). **(F)** Percentage of cells migrating upstream from experiments shown in panel **(E) (G)** Cells expressing the indicated gRNAs were allowed to migrate on ICAM-1-coated surfaces, fixed, and stained with fluorescent phalloidin. Representative images, Scale bar: 10 μm. Adapted from Roy et al., *J Cell Sci*, 2020 September 9; 133(17):jcs248328.

Upstream migration is supported only by engagement of ICAM-1 by LFA-1. Previous work has shown that LFA-1/ICAM-1 forms catch bonds—bonds that dissociate less well when a force is applied (Dembo et al., 1988; Zhang et al., 2002; Wojcikiewicz et al., 2006). It is appealing to think that upon the application of a shear force to the cell, the LFA-1/ICAM-1 bonds in the lamellipod would hold on tighter and pin the lamellipod. However, propulsive migration would also require extension and polymerization of the lamellipodial front, which might be facilitated by outside-in signaling of integrins. β2 integrins are known to signal both ERK and phosphoinositide-3 kinase (PI3K) pathways (Roy et al., 2020), so it seems plausible that LFA-1 could simultaneously act as a catch bond and promote actin polymerization through outside-in signaling. This hypothesis fits with the observation reported by Roy *et al* that T cells migrating on ICAM-1 coated surfaces had a clear lamellipodial structure with robust actin polymerization. (Roy et al., 2018; Roy et al., 2020). Roy and coworkers had previously shown that an adaptor protein, Crk, was a key intermediate downstream of LFA-1 and facilitated the response of T cells to substrate stiffness, another mechanosensitive response (Roy et al., 2018). In a study led by Janis Burkhardt’s laboratory at the Children’s Hospital of Philadelphia, it was found that Crk as well as the ubiquitin ligase c-Cbl, downstream of LFA-1 but before PI3K (Figure 2A), were critical for promoted upstream migration. Activated murine T-cells from Crk double knock-out (DKO) mice reversed the direction of migration under hydrodynamic flow on ICAM-1 surfaces, without affecting actin polymerization and formation of a lamellipod (Figure 2) (Roy et al., 2020). Complementary experiments involving CRISPR Cas9 editing of T-cells from a Cas9 mouse showed that deletion of c-Cbl also reversed the direction of T-cell migration on ICAM-1 (Roy et al., 2020). Interestingly, PI3K itself—either in response to CRISPR deletion or pharmacological inhibition (Dominguez et al., 2015; Roy et al., 2020) - did not affect upstream migration, which suggests there are other key molecules that are affected by Crk and c-Cbl. Further research is needed to identify other molecular components responsible for upstream migration, perhaps by a large scale CRIPSR screen which has been successfully pursued to identify mediators of motility in HL-60 and T-cells (Belliveau et al., 2022; Johansen et al., 2022).

## Crosstalk among integrins

Two T-cell ligands are expressed on the apical surface of endothelium, ICAM-1 and VCAM-1; these are recognized by the integrins LFA-1 and VLA-4, respectively. Theodoly and colleagues had shown that on ICAM-1 surfaces, T-cell displayed upstream migration only when flow was initiated; otherwise, T-cells moved at random (Valignat et al., 2013). Another study furthered this observation by demonstrating that while murine T cells crawled upstream on ICAM-1, they crawled downstream on ICAM-2 and VCAM-1 surfaces on the Blood Brain Barrier (BBB) Microvasculature. (Steiner et al., 2010). Interestingly, while ICAM-2 did not support upstream migration on murine BBB microvasculature cells, T cells did crawl upstream on both purified ICAM-1 and ICAM-2 FC substrates (Steiner et al., 2010) leading to whether the specific biology of the BBB microvasculature inhibited ICAM-2 mediated upstream migration.

To address the role of VCAM-1 on upstream migration, our laboratory made surfaces by coating protein A/G and mixtures of soluble ICAM-1 and VCAM-1 Fc chimeras to alter the ratio of ICAM-1 to VCAM-1. We found that only a small amount of ICAM-1 (10% of all surface ligands) was necessary to support upstream migration in T-cells (Dominguez et al., 2015). By patterning different densities of adhesive ligands in a gradient, Xuo and coworkers showed that T-cells display a preference in adhesiveness depending on the ligand under static conditions (Luo et al., 2020). T-cells display reverse haptotaxis on ICAM-1, crawling towards lower densities of ICAM-1, and haptotaxis towards higher densities of VCAM-1. While the reverse haptotaxis of T cells on ICAM-1 is an interesting biophysical phenomenon, it may not be physiologically relevant as under shear flow conditions ICAM-1 localizes at higher concentrations upstream of the flow direction on endothelial cells (Piechocka et al., 2021). Coupled with the demonstrated propensity of T-cells to migrate upstream on ICAM-1 surfaces, the propensity of T cells to perform reverse haptotaxis on ICAM-1 may be overridden by the signals generated when exposed to shear flow on the vascular endothelium.

In addition, our laboratory did a series of experiments that illustrated that the signals from both integrins are integrated complexly. First, we showed that on surfaces in which the two ligands ICAM-1 and VCAM-1 are mixed 50/50, activated CD4^+^ T cells retained persistence and continued to migrate in the upstream direction, even when the flow was stopped, which we describe this effect as “migrational memory”. In contrast, T cells possessed no memory of upstream migration on 100% ICAM-1 surfaces when the flow was stopped. These results led our laboratory to do an extensive study of the mechanisms of migrational memory (Kim and Hammer, 2019). Starting with the result that activated CD4^+^ T-cells on 50/50 ICAM-1/VCAM-1 surfaces migrated upstream for at least 30 min after shear flow of 8 dynes/cm^2^ was stopped, we used pharmacological inhibitors to show that migrational memory required PI3K, but not the GTPase cdc42 and or the actin branching molecule Arp2/3 (Kim and Hammer, 2019). We showed that while LFA-1 is essential for upstream migration, VLA-4 is essential for the migrational memory. Using a blocking antibody against VLA-4, we also blocked migrational memory in T cells after migrating upstream on VCAM-1/ICAM-1 polyacrylamide gels, independent of surface stiffness and dependent only on the shear flow rate (Kim and Hammer, 2021). On surfaces that lacked VCAM-1, we could promote migrational memory on ICAM-1 surfaces using soluble VCAM-1 to ligate VLA-4 (Kim and Hammer, 2019; Kim and Hammer, 2021). This study implies that the simultaneous activation of LFA-1 and VLA-4 integrins could also propagate actin reorganization to support the upstream migration of T cells. While LFA-1 alone is sufficient to promote upstream migration, we have shown that VLA-4 and LFA-1 together are essential for directed motion of CD4^+^ T lymphocytes post flow. So, while the upstream direction is determined by LFA-1, the persistence of the upstream direction is maintained by simultaneous VLA-4 activation and consequent PI3K signaling generated from the crosstalk of the two integrins in CD4^+^ T cells.

**FIGURE 3:**
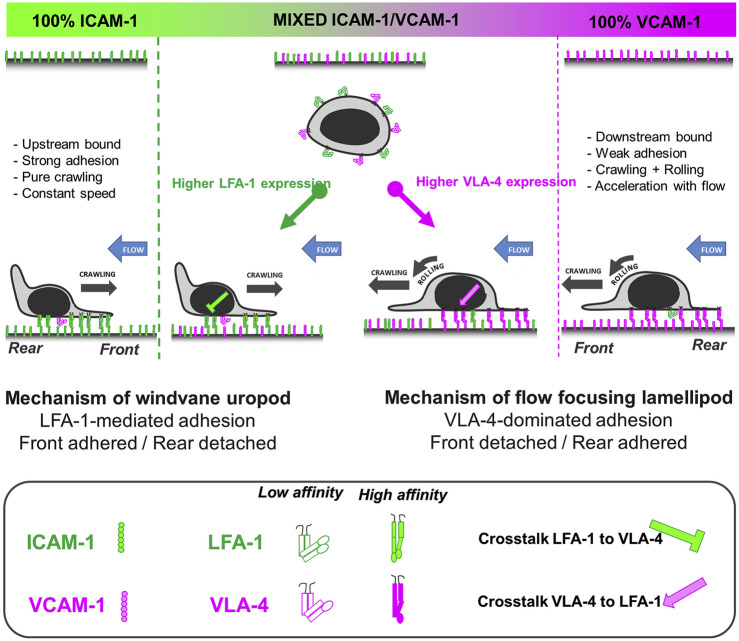
A bistable mechanism of cell adhesion spatial regulation explains integrin control of T cell flow mechanotaxis. On pure substrates of ICAM-1 or VCAM-1, the T cell population has homogeneous phenotypes with an opposite orientation on ICAM-1 and VCAM-1. On mixed substrates of ICAM-1 or VCAM-1, T cells distribute in two populations with opposite orientations and characteristics similar to phenotypes on pure substrates. Decisions of orientation on mixed substrates are controlled by the expression level of integrins LFA-1 and VLA-4 via a bistable polarization of cell adhesion; a higher LFA-1 expression leads to a LFA-1-dominated adhesion of cell front (very similar to upstream crawling cells on ICAM-1), whereas a higher expression of VLA-4 leads to adhesion of cell rear and center (very similar to downstream crawling cells on VCAM-1). Inhibiting cross talk of LFA-1 toward VLA-4 reinforces adhesion polarization toward cell front, which favors wind vane mechanism and upstream phenotype. Activating cross talk of VLA-4 toward LFA-1 reinforces the adhesion of cell uropod, which hampers the wind vane mechanism and favors the downstream phenotype. Adapted from Hornung et al., *Biophys J*, 2020 February 4; 118(3):565-577.

Hornung et al. further investigated the crosstalk of LFA-1 and VLA-4 by utilizing pure ICAM-1, VCAM-1, and molar mixtures of the two on the surface to characterize the migration profiles of T cells (Hornung et al., 2020). They reported that LFA-1-ICAM-1 interactions imposed strong adhesion and upstream migration while VLA-4-VCAM-1 interactions led to transient interactions and downstream migration. Furthermore, they demonstrated that cells attached at the front and were detached at the rear (i.e., lamellipods and uropods, respectively) when migrating upstream, while the opposite (front-detached and rear-adhered) was true during downstream migration (Figure 3) (Hornung et al., 2020). Subsequent studies revealed that active VLA-4 was localized towards the rear of the cell while active LFA-1 populated towards the front (Robert et al., 2021).

## Upstream migration observed in other cell types

The phenomenon of T cells migrating against the direction of shear flow from a fundamental biophysical standpoint is exciting but given the pervasiveness of LFA-1/ICAM-1 interactions, one might question whether other immune cell types might exhibit upstream migration. The following sections summarize the evidence that immune cell types, other than T cells, can migrate upstream against the blood flow, either in vasculature or also in organs and tissues under interstitial flow.

Hematopoietic Stem and Progenitor Cells: Hematopoietic Stem and Progenitor cells (HSPCs) are a heterogeneous mixture of long and short term hematopoietic stem cells (HSCs) and multipotent progenitors (MPPs) of varying degrees of differentiation, defined by the expression of CD34 (Carvalho et al., 2009). Residing in the bone marrow, HSPCs can differentiate into all mature blood cells of both the myeloid and lymphoid origins (Bujko et al., 2019). HSPCs are also found in circulation and able to crawl along the endothelial surface to traffic back to the bone marrow niche (Mazo et al., 1998). The homing and proper trafficking of HSPCs to the bone marrow is considered to be required during HSPC transplantation to restart homeostasis after an ablative chemotherapy (Appelbaum, 2007).

Similar to T cells, HSPCs also express LFA-1 (α_L_β_2_) and VLA-4 (α_4_β_1_) in sufficient levels to confer binding to ICAM-1 and VCAM-1 surfaces (Buffone et al., 2018), which implies potential for HSPCs to also migrate upstream. Like T cells, the HSPCs and KG1a cells we tested did not express Mac-1 (α_M_β_2_), which competes with LFA-1 for ICAM-1 binding. To explore the upstream migration of HSPCs, we decided to use KG1a cell line, an immortalized human cell line widely used as a model for CD34^+^ HSPCs (AbuSamra et al., 2017), to investigate the upstream migration profile in HSPCs. Indeed, KG1a cells migrated upstream on recombinant ICAM-1 surfaces and downstream on VCAM-1 surfaces (Buffone et al., 2018), similar to the of behavior T cells. Further experiments demonstrated that upstream migration required LFA-1-ICAM-1 interactions and was observed on both mixed surfaces of ICAM-1 and VCAM-1, and monolayers of stimulated human umbilical vein endothelial cells (HUVECs) (Buffone et al., 2018). Finally, primary CD34^+^ HSPCs isolated from bone marrow were confirmed to have similar integrin expression profiles to CD4^+^ T cells and KG1a cells and exhibited upstream migration on ICAM-1, mixed ICAM-1 + VCAM-1 surfaces, and HUVEC monolayers. This study highlights that the upstream migration is not limited to T cells and can be extended to HSPCs (Buffone et al., 2018). Studying upstream migration in HSPCs may be used to improve the speed of HSPCs homing to the bone marrow and restarting hematopoiesis after transplantation, especially since the T cells have been shown transmigrate twice as fast migrating upstream than downstream (Anderson et al., 2019).

Marginal Zone B cells: Marginal zone B cells (MZBs), another lymphoid cell type that surveys blood from the circulation as it enters the spleen for antigens (Lopes-Carvalho et al., 2005), were demonstrated to migrate against the direction of shear flow (Tedford et al., 2017). Shear flow in the spleen runs from the follicle, through the marginal zone, and finally into the red pulp. The direction of flow within the spleen requires MZBs to migrate upstream from the marginal zone to deliver antigens to the follicle (Mebius and Kraal, 2005; Cerutti et al., 2013). Splenic endothelial cells express both ICAM-1 and VCAM-1 (Ulyanova et al., 2005), and the shear flow acts as a mechanotransducer to activate both LFA-1 and VLA-4 on the MZBs. MZBs crawl upstream on ICAM-1 and mixed ICAM-1/VCAM-1 surfaces and downstream on VCAM-1 surfaces (Tedford et al., 2017; Tedford et al., 2018). The expression of CXCL13 and Mucosal Vascular Addressin Cellular Adhesion Molecule (MAdCAM-1) in blood vessels retards the upstream migration. Upstream crawling is completely lost when signaling through sphingosine-1-phosphate (S1P) is ablated in knockout mice (Tedford et al., 2017). Interestingly, once the MZBs reach the follicle and become follicular B-cells (FOBs), they lose the ability to crawl upstream (Tedford et al., 2017). These results demonstrate a critical biological function of upstream migration as MZBs must utilize this mode of motility to properly deliver antigen to the follicle.

**FIGURE 4:**
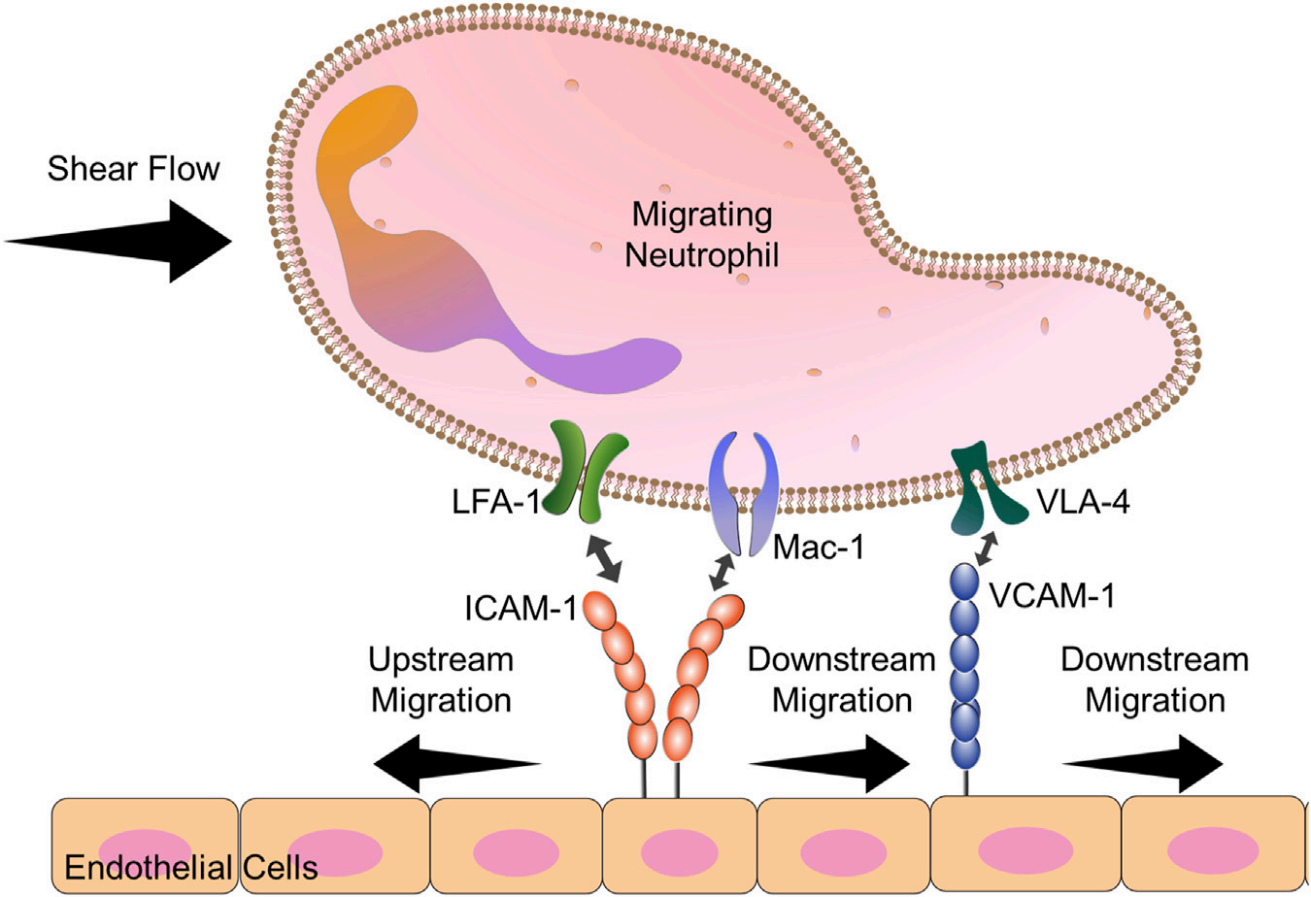
Diagram explains the key players in neutrophil upstream migration. VLA-4-VCAM-1 interactions lead always to downstream migration. Neutrophils have 2 cell surface ligands for ICAM-1: LFA-1 and Mac-1. When both Mac-1 and LFA-1 are allowed to engage ICAM-1, downstream migration is seen. Blocking Mac-1 function with monoclonal antibodies allows for LFA-1-ICAM-1 mediated upstream migration.

Neutrophils: While identifying that CD4^+^ T cells were able to migrate against the direction of shear flow (Valignat et al., 2013), Theodoly and coworkers also interrogated the directional preference of primary human neutrophils. They showed that neutrophils were unable to migrate upstream, even when an antibody against α_M_ chain was present. Other work had shown that neutrophils could migrate perpendicular to the direction of shear flow (Phillipson et al., 2009). We hypothesized that neutrophils exhibit limited upstream migration because neutrophils are of the myeloid lineage that, in addition to LFA-1 and VLA-4, express Mac-1 (α_M_β_2_) (Langereis, 2013), which competes with LFA-1 for ICAM-1 binding (Smith et al., 1989). Indeed, work from our laboratory showed that by varying the amount of ICAM-1 on the surface, both the HL-60 neutrophil-like cell line and primary human neutrophils isolated from whole blood could migrate upstream in ICAM-1 surfaces when Mac-1 function was blocked (Buffone et al., 2019). Blocking Mac-1 allowed both resting and N-Formylmethionine-leucyl-phenylalanine (fMLP)-activated neutrophils to crawl upstream on ICAM-1, mixed ICAM-1/VCAM-1 surfaces, and activated HUVEC monolayers (Figure 4) (Buffone et al., 2019). We believe the difference between our results and the results from the Theodoly laboratory is due to differences in ICAM-1 density; we used lower densities of ICAM-1 which seems to have enhanced the mobility of neutrophils (Valignat et al., 2013; Buffone et al., 2019)

A recent paper by Dupuy *et al.* expanded upon the interplay between Mac-1 and LFA-1 in coordinating neutrophil upstream migration (Dupuy et al., 2023). By treating neutrophils with Protein disulfide Isomerase (PDI), Dupuy *et al.* demonstrated that PDI localizes specifically to Mac-1 at the trailing edge of the cell and selectively cleaves disulfide bonds, leading to Mac-1 release of ICAM-1. Furthermore, while the paper states cleavage of Mac-1 at the rear by PDI led to more downstream migration, this was compared to the bound but uncleaved (oxidized) PDI control. Association of PDI with the α_M_ chain, but not cleavage, lead to significant upstream migration when compared to unbound control, similar to the antibody blocking studies designed by Buffone et al. (Buffone et al., 2019). In both studies (Buffone et al., 2019; Dupuy et al., 2023), globally blocking Mac-1 with oxidized PDI or monoclonal antibodies, lead to over 65% of cells crawling upstream. Dupuy and coworkers’ result indicates that the engagement of PDI alone can promote upstream migration in neutrophils.

Both groups show that neutrophils exhibit upstream migration by blocking Mac-1 and opens possibilities of controlling the ability any immune cell to migrate upstream by expressing or removing Mac-1 function. As to what the difference between LFA-1 and Mac-1, two integrins which compete to bind ICAM-1 and share a common β2 integrin chain, are in terms of distinct, outside-in intracellular signals that confer or disrupt upstream migration is an ongoing area and active area of research in our labs.

Macrophages: While no work to date has demonstrated the ability of monocytes to migrate upstream along the endothelium, recent work has demonstrated that extracellular matrix (ECM) resident macrophages are able to migrate against the direction of interstitial flow (Li et al., 2018), not blood flow like the other immune cells. This expands the phenomenon of upstream migration as this is the first demonstration of interstitial flow in the ECM as a mechanic stimulus (Quaranta, 2000) and with a ligand other than ICAM-1. These macrophages migrate upstream on collagen I surfaces, not vascular ICAM-1 and through β1, not β2 integrins like the other three immune cell types.

Interstitial flow emanating from tumors into the surrounding stroma activates mechanosensitive β1 integrins which activate SRC kinases and polarize macrophages into an M2 phenotype (Li et al., 2018). M2 Macrophages then migrate against the direction of interstitial flow from the ECM into the tumor microenvironment (TME). M2 Macrophage are immunosuppressive, actively play a role in shielding tumors from immunosurveillance, and secrete growth factors which make the tumor more invasive (Li et al., 2018). Furthermore, both MDA-435 melanoma cells and MDA-231 breast cancer cells were more protrusive and migratory when co-cultured with macrophages which had been exposed to interstitial flow (Li et al., 2018). Therefore, upstream migration by interstitial flow may be a critical regulator of escape from immunosurveillance by both polarizing and recruiting M2 macrophages into the tumor microenvironment and tumor progression and metastasis by allowing tumor cells to escape into the vasculature. It should be noted that the mechanism of M2 macrophage movement likely differs compared to other immune cell types, due to its abundant expression of Mac-1, but this remains an open area of investigation as to the direct mechanism.

Keratinocytes: Recent work was the first to establish that the upstream migration mode of motility is not restricted only to immune cells as a certain sub-population of fish keratinocytes are able to crawl upstream on glass slides (Seveau de Noray et al., 2022). Although the keratinocytes preferentially migrated downstream, a sub-population with a prominent trailing edge were able to crawl upstream, as compared to those with a prominent leading-edge.

The results regarding upstream migration in other cell types are summarized in Table 1.

**TABLE 1:** Cell types demonstrating upstream migration.

Cell type	Primary cells and cell lines	Mac-1 expressed	Mac-1 blocking needed?	References
T cells	Primary CD4^+^ and CD8^+^ (human and murine)	NO	NO	Bartholomaus et al. (2009), Steiner et al. (2010), Valignat et al. (2013), Valignat et al. (2014), Dominguez et al. (2015), Anderson et al. (2019), Hornung et al. (2020), Roy et al. (2020), Piechocka et al. (2021)
Hematopoietic Stem and Progenitor cells	KG1a (cell line) and Primary (human)	NO	NO	Buffone et al. (2018)
Marginal Zone B cells	Primary (murine)	NO	NO	Tedford et al. (2017)
Neutrophils	HL-60 (cell line) and Primary (human whole blood)	YES	YES	Buffone et al. (2019), Dupuy et al. (2023)
Macrophages	Raw 264.7 (cell line) and primary (human bone marrow derived)	YES	Interstitial Flow-NO	Polacheck et al. (2014), Li et al. (2018)
Keratinocytes	Primary (Fish)	NO	NO	Quaranta (2000)

## The physiological role of upstream migration

There are at least three possibilities for the role of upstream migration in physiology. The first, suggested by many, is that upstream migration might be required for immune cells to return to the site of stimulation or insult, a so called “hotspot” (Bartholomaus et al., 2009; Valignat et al., 2013; Grönloh et al., 2023). The second, suggested in a recent seminar by Ulrich H. von Andrian, shows that memory T-cells migrate upstream and this might facilitate the repeated surveillance of a tissue as both effector and memory T cells were observed to repeatedly migrate upstream (Von Andrian, 2022). The third possibility is that upstream migration might facilitate transendothelial migration (TEM), because the mechanobiological forces exerted in upstream migration might be similar to those that are observed in migrating to the right junction for TEM to occur.

**FIGURE 5:**
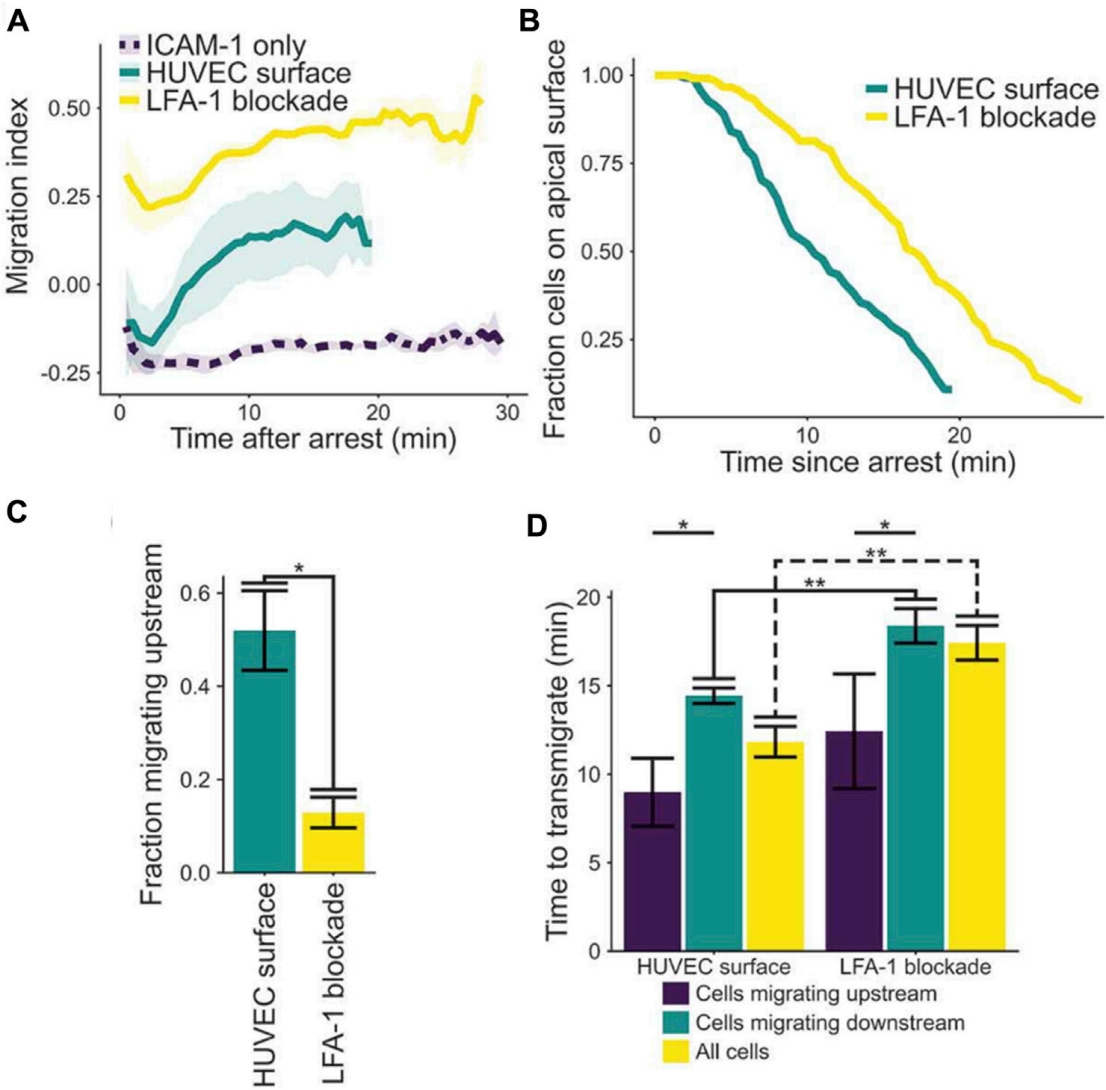
**(A)** Plot of migration index over time of activated CD4^+^ T cells on activated HUVEC and ICAM-1 surfaces. Upstream migration is indicated by a negative migration index, downstream migration by positive values, and random migration by values near zero. Blockade of LFA-1 prevents upstream migration on stimulated HUVECS, while cells with unblocked LFA-1 initially migrate upstream before reverting to downstream migration. ICAM-1-only recombinant protein surface data is provided for comparison. Data presented mean ± SEM, n = 4 independent experiments. **(B)** Plot showing the remaining fraction of tracked cells at each time point. Cells on HUVEC monolayers were tracked from initial migration to transmigration or the end of the experiment, whichever is sooner. **(C)** Comparison of fraction of cells which migrated upstream on HUVEC monolayers with or without LFA-1 blockade. Data presented mean ± SEM, n = 4 independent experiments. **(D)** Comparison of the time from arrest to transmigration on HUVEC monolayers with or without LFA-1 blockade. Data presented mean ± SEM, n = 4 independent experiments. *p < 0.05, **p < 0.005. Reproduced from Anderson et al., *Cell Adh Migr,* 2019 December; 13(1):163-168.

To support of the third concept, Anderson, Buffone & Hammer demonstrated upstream migration of T cells on activated human umbilical vein endothelial cells (HUVECs) monolayers (Anderson et al., 2019). T cells were observed to initially migrate upstream after arrest, provided LFA-1 was not blocked; however, the initial population of upstream migrating cells transmigrated (Figure 5; (Anderson et al., 2019)). The fraction of cells that remained on the apical surface of endothelium was greater if LFA-1 was blocked, suggesting LFA-1 facilitated transendothelial migration. Also, if cells were able to migrate upstream, the time to transmigrate was greatly diminished (Figure 5). These results suggest LFA-1 can mediate both transendothelial migration and upstream migration, and that the mechanisms that support upstream migration increase the speed and extent of transendothelial cell migration (Anderson et al., 2019).

## Future work

The upstream migration of leukocytes is a fascinating biophysical phenomenon. Cells actively migrate against the direction of flow, which requires harvesting mechanical and chemical machinery. Flow imparts a body force on cells, and cells must overcome that body force to stay adhered, detect the direction of flow, and reorient themselves to move in the opposite direction. Furthermore, what physiological mechanism/process makes it critical that lymphoid cells (T cells, B cells) readily migrate upstream while myeloid cells (neutrophils) do not unless “coaxed” to by antibody blocking is of great interest.

Accurate measurements of the forces generated during upstream migration can lead to a greater understanding of the phenomenon of the whole. Traction mapping through use of either traction force microscopy or multi-post array detectors (mPADs) have been used to generate traction maps previously for a variety of migrating immune cells (Dembo et al., 1996; Smith et al., 2007; Reinhart-King et al., 2008; Jannat et al., 2011; Ricart et al., 2011; Shebanova and Hammer, 2012; Henry et al., 2015; Hind et al., 2015; Hind et al., 2016; Bendell et al., 2017). It has proven difficult to determine traction maps of T-cells (Nordenfelt et al., 2016), as the force they exert is at the lower limit of detection in these assays. But upstream migration is not restricted to T cells, and HL-60 cells, which are often thought to be “neutrophil-like” (Garner et al., 2020), are able to readily migrate upstream on ICAM-1 surfaces where after Mac-1 is blocked (Buffone et al., 2019). Traction forces of neutrophils are readily measurable though bead displacement of embedded marker beads in elastic gels (Smith et al., 2007; Jannat et al., 2011). Interestingly under static conditions, the root mean squared (r.m.s) traction stress for neutrophils is about 50 nN; in chemotaxis, the r.m.s traction stress can be as high as 100 nN (Jannat et al., 2011). Therefore, one would expect traction stresses as high as 50 nN or higher for HL-60 cells or neutrophils that are migrating upstream. Moving forward, neutrophils (both HL-60 and primary neutrophils from whole blood) are viable cells to measure the traction stresses during the upstream migration owing to the ability of their traction forces to be measured (Smith et al., 2007; Jannat et al., 2011), and the ease to which they can be genetically manipulated (Buffone et al., 2013; Mondal et al., 2014; Mondal et al., 2016; Stolfa et al., 2016; Kelkar et al., 2020; Zhu et al., 2021).

An ongoing question is whether immune cells in the vasculature migrate upstream outside of the vasculature. The work of the Kamm lab in macrophage polarization against the direction of interstitial flows (Polacheck et al., 2011; Polacheck et al., 2014) raises the interesting question of whether immune cells also respond to interstitial flow, and migrate in the opposite direction after transmigration, and whether this mechanism can be controlled to promote more efficient cell migration through tissues.

Further investigation of the physiological role of upstream migration is needed. Since most data describing this phenomenon has been done in *in-vitro* assays (Valignat et al., 2013; Dominguez et al., 2015; Tedford et al., 2017; Buffone et al., 2018; Anderson et al., 2019; Buffone et al., 2019; Roy et al., 2020), more careful studies into the specific context in which cells migrate upstream *in-vivo* is needed. Some studies have shown a random migration of immune cells *in-vivo* (Phillipson et al., 2006; Phillipson et al., 2009; Snelgrove et al., 2019; Kibaek et al., 2021) while others have shown prominent upstream migration *in-vivo* (Bartholomaus et al., 2009; Steiner et al., 2010). The next step of truly understanding the importance of upstream migration will be in which physiologically relevant situations it occurs. Such an *in-vivo* test would involve an animal disease model for immune cell surveillance and clearance, in which one can compare the physiological response of cells which are able to migrate upstream (wild type T cells) and those in which upstream migration is disabled (edited T cells). The work of Roy et al. points to candidate molecules that can be depleted to disable upstream migration (Roy et al., 2020), but wider screens may be necessary to identify specific molecules that enable upstream migration without affecting other physiological responses. Such experiments would also be aided by *in vivo* fluorescent tracking of the differential response of immune cells in a disease model (John et al., 2011).
